# Declining number of home visits to older adults by GPs: an observational study using data from electronic health records in The Netherlands, 2017–2023

**DOI:** 10.3399/BJGPO.2024.0255

**Published:** 2025-06-18

**Authors:** Chantal J Leemrijse, Marianne J Heins, Bart J Knottnerus, Mariette Hooiveld, Judith N de Boer, Ron F Schipper, Joost W Vanhommerig

**Affiliations:** 1 Nivel, Netherlands Institute for Health Services Research, Utrecht, The Netherlands; 2 Dutch Healthcare Authority (NZa), Utrecht, The Netherlands

**Keywords:** general practitioners, home visits, house calls, electronic health records

## Abstract

**Background:**

Despite an ageing population that has higher care demands, home visits by GPs have been declining.

**Aim:**

To analyse the number of GP home visits from 2017–2023 in The Netherlands, and to investigate whether this trend differed according to age, sex, multimorbidity, and neighbourhood deprivation. In addition, to discover the most common reasons for home visits between 2017 and 2023.

**Design & setting:**

An observational study that used data derived from Nivel Primary Care Database (Nivel-PCD), which contained routinely recorded data from approximately 500 Dutch GP practices.

**Method:**

The number of home visits was calculated by age, sex, multimorbidity, and neighbourhood deprivation. Visual inspection was used to investigate the relation between the trend in number of home visits and age, sex, multimorbidity, and neighbourhood deprivation of patients.

**Results:**

A large decrease in the overall number of home visits was observed between 2017 and 2023 (-32%). The largest decrease was between 2019 and 2020 (-15%), but the downward trend continued in 2021 through 2023 (-5% per year). The most profound decline between 2023 and 2017 was found in the number of short home visits (-52%). The number of home visits for intensive GP care increased by 12% between 2017 and 2023.

**Conclusion:**

We report a continuing decline in the number of home visits by GPs, comparing 2023 with 2017. Home visits for intensive GP care, often for patients at the end of life, increased since 2017. GPs may be forced to make choices owing to the increasing workload.

## How this fits in

Before this research, a decline in GP home visits in The Netherlands was already observed, and a further strong decrease occurred during the COVID-19 pandemic. It was unclear whether this decline would persist after the pandemic-related restrictions eased. This study reports on data from before, during, and after the COVID-19 pandemic, demonstrating that the decline in GP home visits has been sustained from 2017–2023. This finding is relevant for clinicians and policymakers, suggesting a potential shift in healthcare delivery and highlighting the need to understand the factors influencing this trend.

## Introduction

Home visits by GPs are a service for patients who are homebound and have difficulty getting to, or are unable to visit the GP practice. Home visits are made in acute medical situations, but can also be scheduled as a routine part of regular GP care for patients with chronic conditions or for those with a social reason, such as social isolation. They may also be important as a part of end-of-life care for severely ill patients.^1^

Although home visits demand a lot of time from already (over)burdened GPs, they have documented benefits, especially for older adults. During routine home visits to older adults, the GP may discover health and/or social problems that can still be resolved in time to prevent aggravation.^2,3^ Furthermore, GP home visits have indeed been related to fewer emergency room and hospital admissions.^4^ Finally, GPs can play an important role in the provision of palliative care for terminally ill patients, and home visits may enable terminally ill patients to die at home instead of in the hospital or another institution.^5,6^ Despite an ageing population, home visits by GPs have been declining worldwide for several decades.^1,7–10^ In Australia, the number of home visits by GPs has more than halved in the past decade^11^ and in both Republic of Ireland and the Swiss canton of Vaud, the absolute number of home visits fell by 40% in 10 years;^12^ the same trend was noticed in Germany.^13^ There may be several reasons for the decline in home visits. In The Netherlands, the number of home visits by GPs fell substantially during the first year of the COVID-19 pandemic,^14^ while the number of home visits had been declining for over a decade already.^15,16^

In 2020–2021, the COVID-19 pandemic further accelerated the decline in home visits. Face-to-face contact with patients was avoided, scheduled care for chronic diseases was mostly postponed,^17,18^ and the number of home visits decreased sharply.^19–22^ It made sense to reduce the number of non-urgent home visits; especially during the first wave of the COVID-19 pandemic, when GPs had limited personal protective equipment and their vulnerable patients were at high risk of COVID-19 infection.^23^ However, severely ill patients continued to require palliative or end-of-life care from their GP, and also patients with COVID-19 with serious health problems needed care.^24^

The question arises whether this accelerated decline heralds the end of ‘medically less urgent’ home visits by the GP in The Netherlands. Therefore, the first aim of our study was to analyse the number and type (that is, short, long, or intensive GP care) of home visits from 2017–2023. The second aim was to investigate possible associations between the observed trend in home visits with age, sex, comorbidity, and socioeconomic position (SEP), as previous studies showed that the number of home visits is associated with these factors.^13,25–29^ Because home visits are most frequently made to older adults, we restricted our analyses to people aged≥65 years. In addition to the number of home visits, the most common reasons for home visits were analysed between 2017 and 2023.

## Method

### Nivel Primary Care Database

Data for this study were derived from Nivel Primary Care Database (Nivel-PCD).^30^ Nivel (the Netherlands Institute for Health Services Research) collects longitudinal data that are routinely recorded by GPs in electronic health records and processes these data into Nivel-PCD. Nivel-PCD contains data on the number and type of contacts with the GP, diagnoses recorded during these contacts (based on ICPC-1; International Classification of Primary Care),^31^ medical prescriptions, and referrals to secondary care. Currently, data are collected from a dynamic cohort of approximately 500 practices (representing about 10% of the Dutch population) spread throughout The Netherlands. Patients in Nivel-PCD are a representative sample of the Dutch population as to age and sex.^14^

### Registration fee

Nearly all Dutch inhabitants are registered with a GP and healthcare insurance is obligatory. GPs receive a quarterly registration fee for each enlisted patient from its healthcare insurance company. This registration fee is higher for older adults and for patients from deprived neighbourhoods.^32^ These so-called deprived neighbourhoods have an above-average share of residents with either a low income, a non-Western or Central or Eastern European migration background, or unemployment. The definition of deprived area is based on postal code and is subject to change as the composition of inhabitants in a postal code may change over time. Residents living in these districts more often have multiple chronic conditions; for example, high blood pressure, diabetes mellitus, chronic obstructive pulmonary disease (COPD), and therefore have more frequent contact with the GP. Data about registration fees are also available within Nivel-PCD and they were used to determine when patients are registered and whether they are living in deprived neighbourhoods. Living in a deprived neighbourhood was used as a proxy for SEP.

### Home visits

For this study, the number of general home visits, which were short visits (<20 minutes) and long visits (≥20 minute), and ‘home visits for intensive GP care’ were studied based on performance codes. The latter are mostly home visits for terminally ill patients, but from March 2020 until December 2022 this performance code could also be used for home visits for (palliative and non-palliative care for) patients with COVID-19.

### Statistical analyses

For each of the years 2017–2023, we calculated the number of visits per 1000 person-years, separately for general home visits and home visits for intensive GP care. Person-years of follow-up were calculated as the sum of time (that is, quarters) each patient was registered in the GP practice. The number of home visits were analysed by patient characteristics age (65–79 years, ≥80 years), sex (male or female), living in a deprived neighbourhood (yes or no), and multimorbidity (yes or no). Multimorbidity was defined as having two or more chronic diseases.^33^

## Results

### General characteristics

The characteristics of the study population are presented in Table 1. Overall, patients aged ≥80 years, women, and patients with multimorbidity received the most home visits (that is, general or intensive GP care) in all calendar years (Supplementary Table S1).

**Table 1.** Study population of older adults (aged ≥65 years) in Nivel Primary Care Database; The Netherlands, 2017–2023

Characteristic	**2017**	**2018**	**2019**	**2020**	**2021**	**2022**	**2023**
Number of patients (*n*)	251 644	233 010	282 206	245 282	252 832	312 696	355 511
Follow-up time (years)	246 410	228 234	276 516	240 740	248 005	306 709	348 751
Female sex (%)	54	53	53	53	53	53	53
Aged 65–79 years (%)	76	76	76	76	76	76	76
Aged ≥80 years (%)	24	24	24	24	24	24	24
Living in deprived neighbourhood^a^ (%)	4	4	5	6	5	5	6
Multimorbidity^b^ (%)	74	75	76	77	77	76	77

^a^Neighbourhood deprivation was determined by the registration fee for the GP. ^b^Multimorbidity was determined as having ≥2 chronic diseases

### Trend in the overall number of home visits

**Figure 1.**
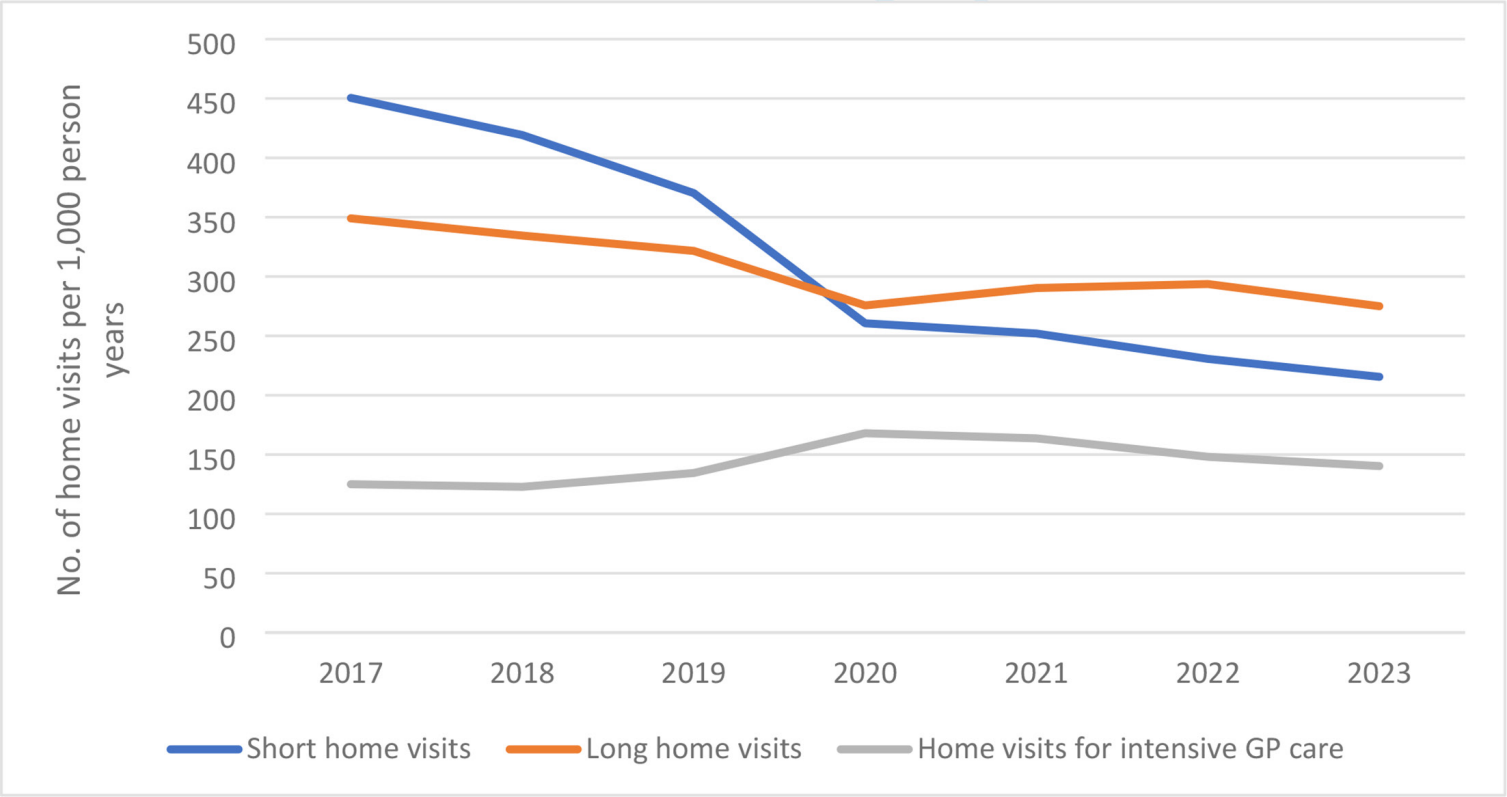
Trends in number of home visits (per 1000 person-years) by GPs to older adults (aged ≥65 years) in Nivel Primary Care Database; The Netherlands, 2017–2023

The overall number of home visits by GPs decreased considerably from 924/1000 person-years in 2017 to 630/1000 person-years in 2023 (-32%; Figure 1 and Supplementary Table S2). The largest decrease was observed between 2019 and 2020, the first year of the COVID-19 pandemic (-15%), but the overall number of home visits continued to decline thereafter with -5% per year. The decrease was most visible in the number of short home visits, with a decline of 52% between 2017 and 2023 (-30% between 2019 and 2020). Long home visits declined with 21% between 2017 and 2023 (-14% between 2019 and 2020). The number of home visits for intensive GP care increased in 2020 (+25%), and although it decreased again in the following years, the number was still higher in 2023 compared with 2019 (+4%).

### Associations of patient characteristics with trends in number of home visits

#### General home visits

The number of general home visits decreased between 2017 and 2023 for all groups of patients, except for females aged 65–79 years living in a deprived neighbourhood (+25%). Apart from this finding, the decrease in number of home visits was irrespective of age, sex, neighbourhood deprivation, or multimorbidity (Table 2a). The decrease in number of home visits appears to be less in deprived neighbourhoods compared with non-deprived neighbourhoods. Only among men aged ≥80 years did the number of home visits decrease evenly between 2017 and 2023 (-49% versus -51% among those without, and -37% versus -37% among those with multimorbidity).

**Table 2.** Relative change in home visits (per cent) by GPs from 2017–2023, by patient characteristics; (a) general home visits and (b) home visits for intensive GP care

		No multimorbidity		Multimorbidity	
		Non-deprived neighbourhood	Deprived neighbourhood	Non-deprived neighbourhood	Deprived neighbourhood
a) General home visits					
65–79 years	Female	-44%	+25%	-40%	-31%
	Male	-42%	-23%	-40%	-28%
≥80	Female	-54%	-26%	-39%	-33%
	Male	-49%	-51%	-37%	-37%
b) Home visits for intensive GP care					
65–79 years	Female	+7%	-39%	+7%	+15%
	Male	-27%	-76%	+0%	+99%
≥80	Female	-18%	-31%	+19%	+28%
	Male	-23%	n.a.^a^	+12%	-6%

Source: Nivel Primary Care Database; The Netherlands. ^a^n.a. = not applicable; the number of home visits in this category was 0 in 2017

#### Home visits for intensive GP care

The number of home visits for intensive GP care increased by 12% between 2017 and 2023 for persons with multimorbidity (Table 2b). These results were irrespective of sex, age, or neighbourhood deprivation, with the exception of males aged ≥80 years, living in a deprived neighbourhood (-6%), and males aged 65-79 years, for whom there was no change (+0%). Among persons with no multimorbidity, there was a decrease in the number of home visits among all categories except females aged 65–79 years living in non-deprived neighbourhoods (+7%).

### Health problems for which home visits are made

The top five most common health problems or diagnoses for which general home visits were made, was relatively stable over the years 2017–2023 (Table 3). General deterioration, heart failure, diabetes mellitus, emphysema or COPD and uncomplicated hypertension were the five most frequently registered reasons for home visits in all calendar years 2017–2023, albeit the order of the top five varied over time.

**Table 3.** Top five of most commonly recorded reasons for home visits, per type of home visit by GPs to older adults (aged ≥65 years) in Nivel Primary Care Database; The Netherlands, 2017–2023

Diagnosis registered for general home visits (Per cent of the total number of diagnoses; min–max)	Diagnosis registered for home visits for intensive GP care (Per cent of the total number of diagnoses; min–max)
General deterioration (3–7%)	General deterioration (5–7%)
Heart failure (3–3%)	Malignant neoplasm bronchus or lung (4–5%)
Diabetes mellitus (3–3%)	End-of-life conversation (4–6%)
Emphysema or COPD (2–3%)	Dementia^a^ (not in top five in 2020–2021) (4–4%)
Uncomplicated hypertension (2–3%)	Heart failure (3–4%)
	COVID-19 infection^b^ (number 4 in 2020 and number 1 in 2021) (4% and 6%, respectively)

^a^Including senile Alzheimer’s. ^b^The diagnosis code recorded was R83, strictly ‘Respiratory infection, other’, this code was used for COVID-19 infection since mid-2020. COPD = chronic obstructive pulmonary disease

Home visits for intensive GP care were mostly performed for patients with general deterioration, malignancies of bronchus or lung, end-of-life conversation, and heart failure. During 2020 and 2021 intensive GP care for COVID-19 infection was (temporarily) in the top five most commonly registered diagnoses, while dementia was one of the five most commonly registered diagnoses in the years before and after the COVID-19 pandemic.

## Discussion

### Summary

The first aim of this study was to analyse the trend in the number of GP home visits between 2017 and 2023. The second aim was to investigate possible associations between this trend and patient characteristics: age, sex, multimorbidity, and neighbourhood deprivation.

We found that the overall number of home visits by the GP decreased by 32% in The Netherlands between 2017 and 2023. During the COVID-19 pandemic in 2020, the number of short and long general home visits dropped substantially by 30% and 14%, respectively. The total number of general home visits was still lower in 2023 than in 2017. In contrast, the number of home visits for intensive GP care, which are mainly directed at end-of-life care, strongly increased in 2020 (that is, by 25%), probably partly owing to severely ill patients with COVID-19. In 2023, the number of these visits was still higher compared with 2017. Other studies have shown a decrease in the number of home visits by GPs during the COVID-19 pandemic,^19–22^ but to the authors’ best knowledge, no studies have been published to date that presented registration data for the subsequent period.

When looking for associations between the observed trend in the number of home visits and patient characteristics, we found that the number of general (short and long) home visits decreased regardless of age, sex, multimorbidity, or neighbourhood deprivation. The only exception were females with no multimorbidity who lived in a deprived neighbourhood, in which the relative amount of home visits increased (+25%). However, the relatively small number of females aged 65–79 years living in deprived neighborhoods complicates the interpretation of this finding.

In general, patients aged ≥80 years patients with multimorbidity, and females were most likely to receive home visits from their GP. This finding is consistent with other studies.^13,28,29^ The number of home visits for intensive GP care increased between 2017 and 2023 for most patient groups, but a decrease was observed among males and females with no multimorbidity. Maybe this latter group is relatively less vulnerable.

### Strengths and limitations

This study is, as far as the authors are aware, the first that uses routinely collected registration data from the years before the COVID-19 pandemic until the last limiting measures were lifted in The Netherlands (2022) and thereafter, to study trends in home visits by GPs. Unfortunately, owing to the claims data we studied, we were unable to distinguish between home visits performed by the GP or a practice nurse. The ratio of patients living in deprived neighbourhoods to patients living in non-deprived was skewed in this study. This made differences between these groups difficult to interpret, especially when other factors such as age and sex were also taken into account. Furthermore, it was not possible to develop a useful multivariable model to analyse results because the considered factors were all heavily interrelated and the number of home visits showed very different trends within subgroups. For this reason, results were based on visual inspection of the data. The reasons for the continuing decrease in the number of general home visits and the increase in home visits for intensive GP care were not investigated in our study and can only be assumed.

### Comparison with existing literature

GP care in The Netherlands currently has to deal with high work pressure and increasing staff shortages, which are reasons likely to contribute to a decrease in performing time-consuming home visits, especially for the medically less urgent visits. One would expect that the ageing population would lead to an increase in demand for home visits, but older adults are also considered to be more mobile. After the COVID-19 pandemic, the number of in-practice consultations continued to increase.^14^ This could lead to a further limiting of the already decreasing number of general home visits by GPs. Telemonitoring may increasingly replace these home visits, except for the most vulnerable population. Unfortunately, we could not study trends for telephone or e-consultations, as these no longer have specific claim codes as of 2019. A German study among 51 GPs in 2021 indicated that longer consultation times, and especially SARS-CoV-2 vaccination consultations, were associated with reduced care services by the practices, including home visits.^22^

### Implications for research and practice

In our study, the number of home visits for intensive GP care increased over time. For this group of patients, continuity of care is very important. An earlier questionnaire study by Plat *et al* ^34^ found that one-third of Dutch GPs were willing to make home visits for severely ill or palliative patients in their private time. This finding is in agreement with other European studies. In a recent survey among 1051 Spanish GPs on what the future (post-COVID-19) work model of GPs should look like, participants stated that home care and end-of-life care remain the responsibility of every GP.^35^

Studies have shown a negative association between home visits and younger age, part-time working, and female sex of the GP.^15,16^ Since both the number of part-time working and the proportion of female GPs are increasing in The Netherlands, one can expect that the number of home visits by GPs will further decrease in the future. However, many patients, especially older adults, consider general home visits to be essential for their wellbeing and the GP–patient relationship, and subsequently for better care.^28,36^ In the context of the current high workload for GPs and the needs and wishes of an ageing population, the integration of other health professionals in home care for older patients and/or those with multimorbidity may be part of the solution.^37,38^ Specially trained nurses can provide care that, in terms of the care process, is at least equivalent to the care provided by physicians for the treatment of chronic diseases.^39^ The role of community services was not part of our study and needs to be studied in more detail.

In conclusion, the number of general home visits is declining in The Netherlands while the number of home visits for intensive GP care increases. Differences in trends in the number of home visits in subgroups of patients with respect to neighbourhood deprivation, age, sex, and multimorbidity were found, but difficult to interpret owing to methodological factors.

## Data

The dataset relied on in this article is available from the corresponding author on reasonable request.
